# Supplementary figures and images for: A reduction in voluntary physical activity in early pregnancy in mice is mediated by prolactin
Source: eLife. 2021 Sep 16;10:e62260. doi: 10.7554/eLife.62260 (PMC8480982; doi:10.7554/eLife.62260)

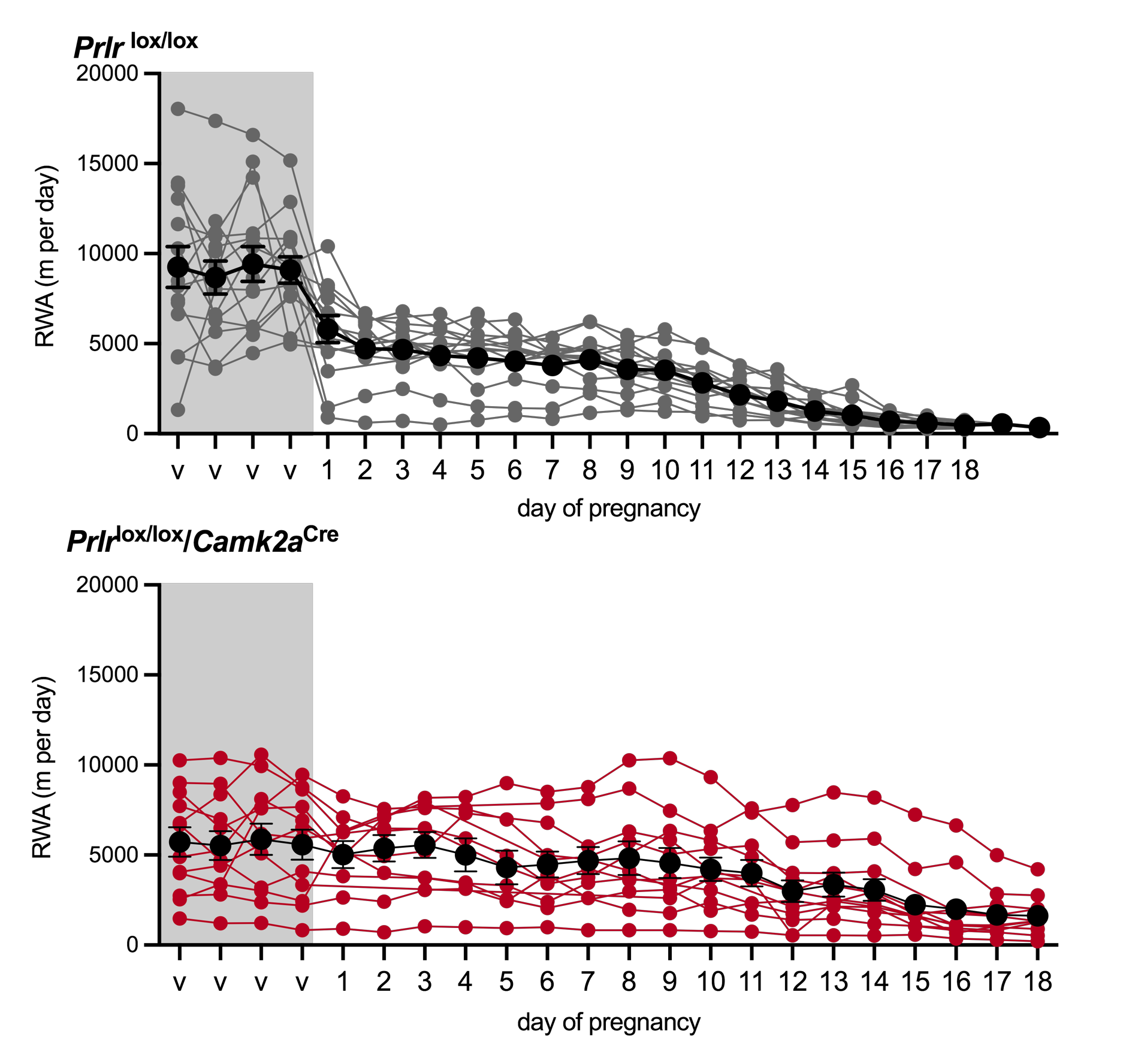

Supplement: Figure 4—source data 1. — Black lines show the mean ± SEM for the group. [file elife-62260-fig4-data1.tif]

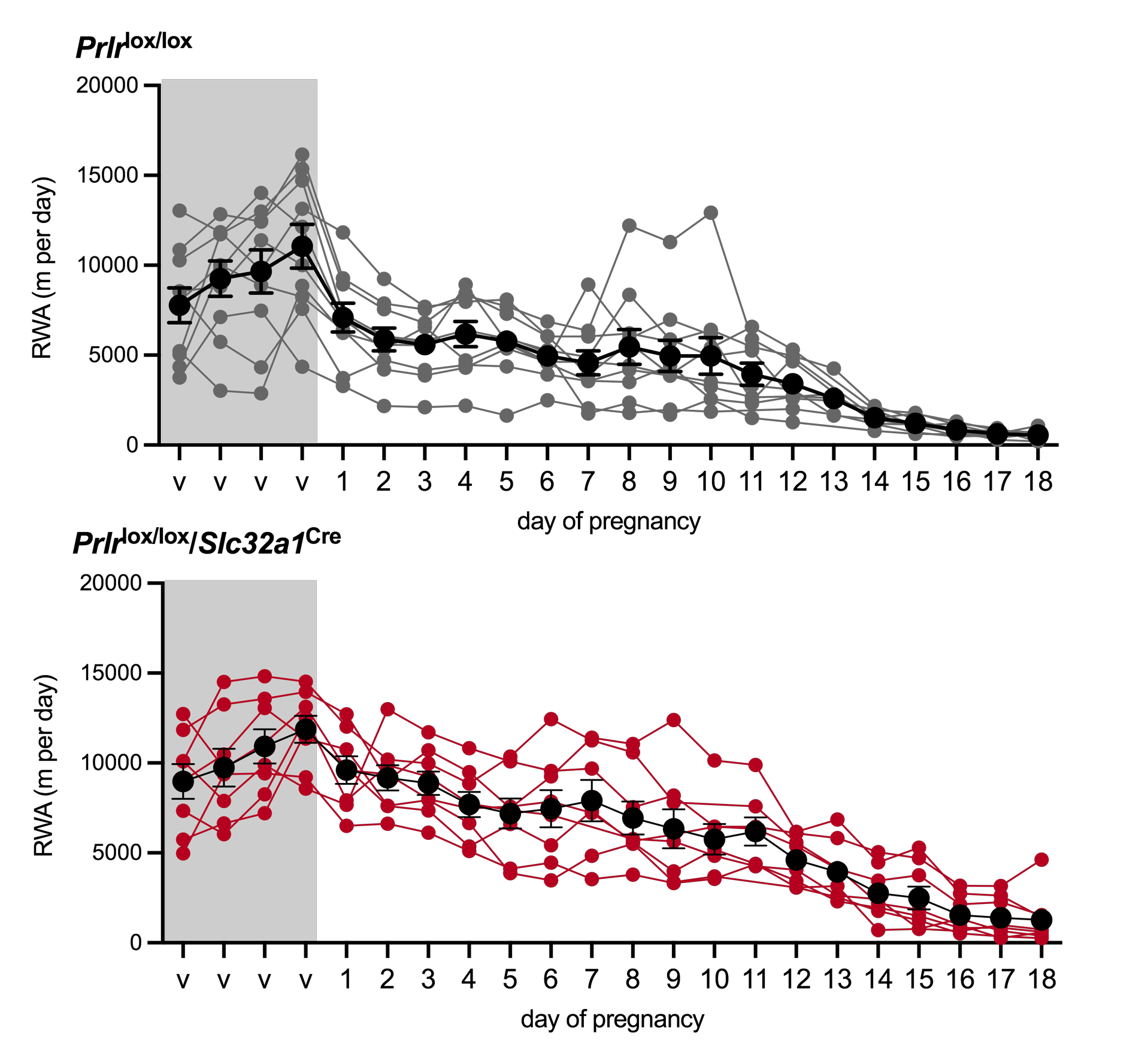

Supplement: Figure 4—source data 2. — Black lines show the mean ± SEM for the group. [file elife-62260-fig4-data2.tif]

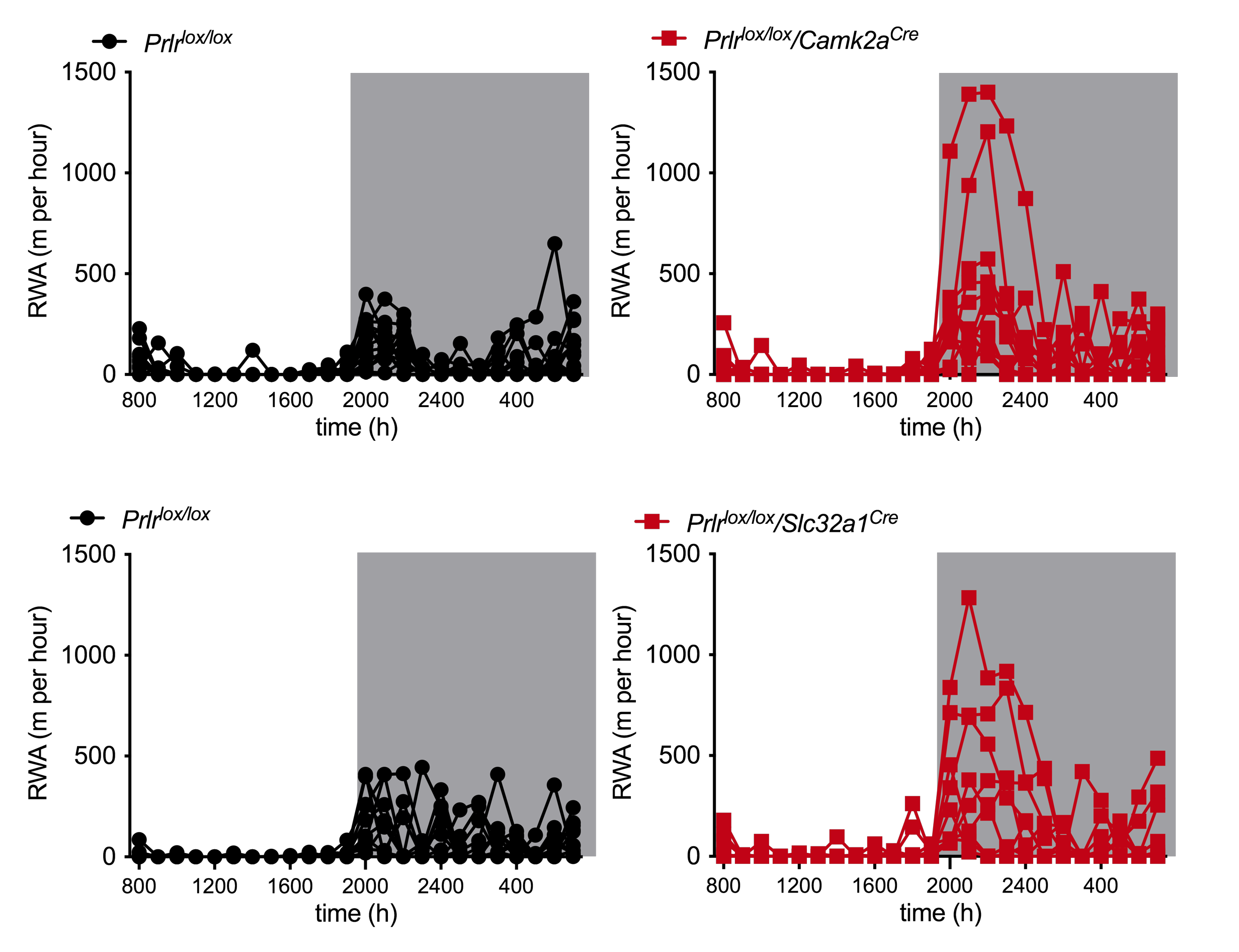

Supplement: Figure 4—source data 3. — Shaded area represents dark phase of the light cycle. [file elife-62260-fig4-data3.tif]

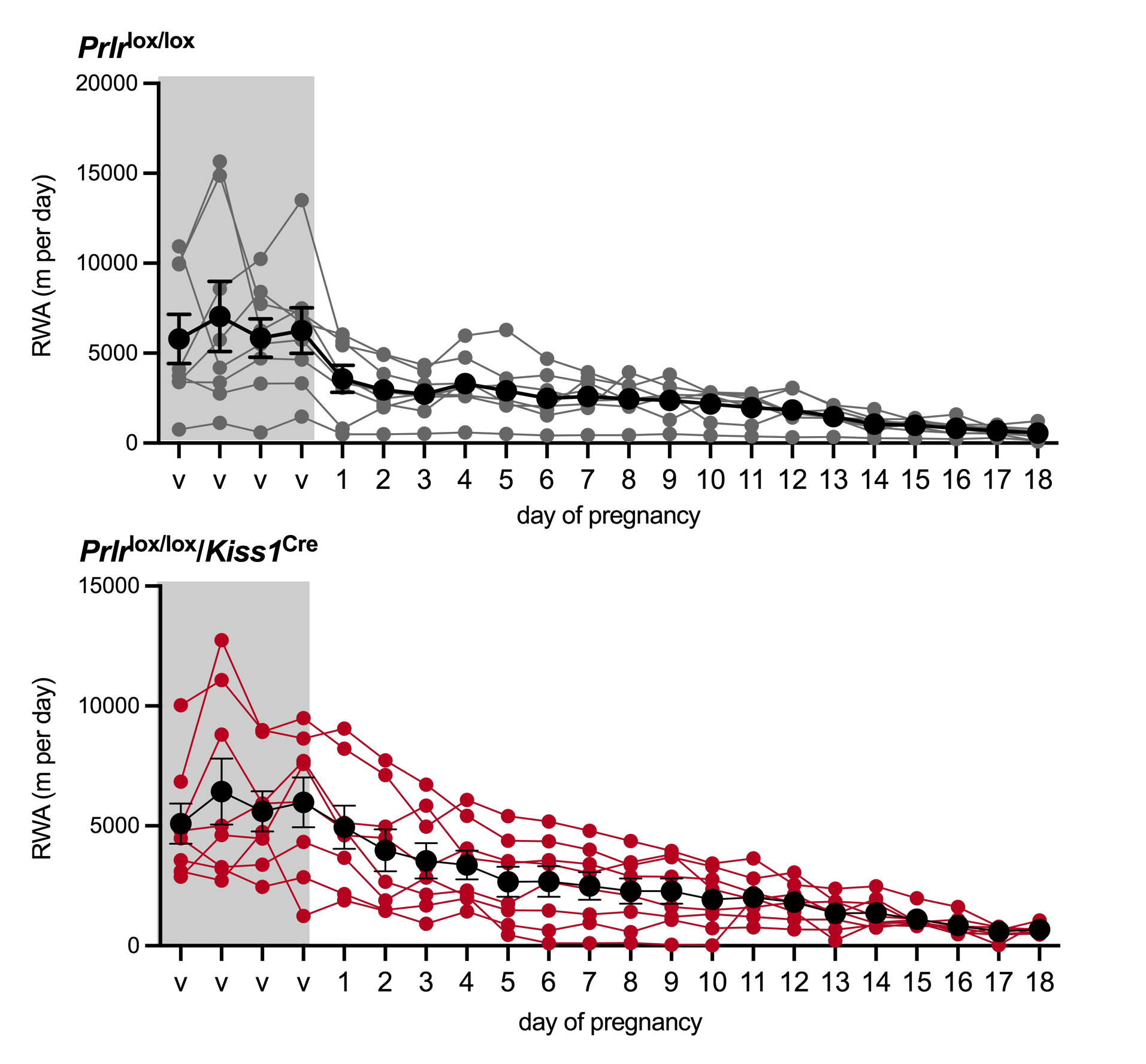

Supplement: Figure 5—source data 1. — Black lines show the mean ± SEM for the group. [file elife-62260-fig5-data1.tif]

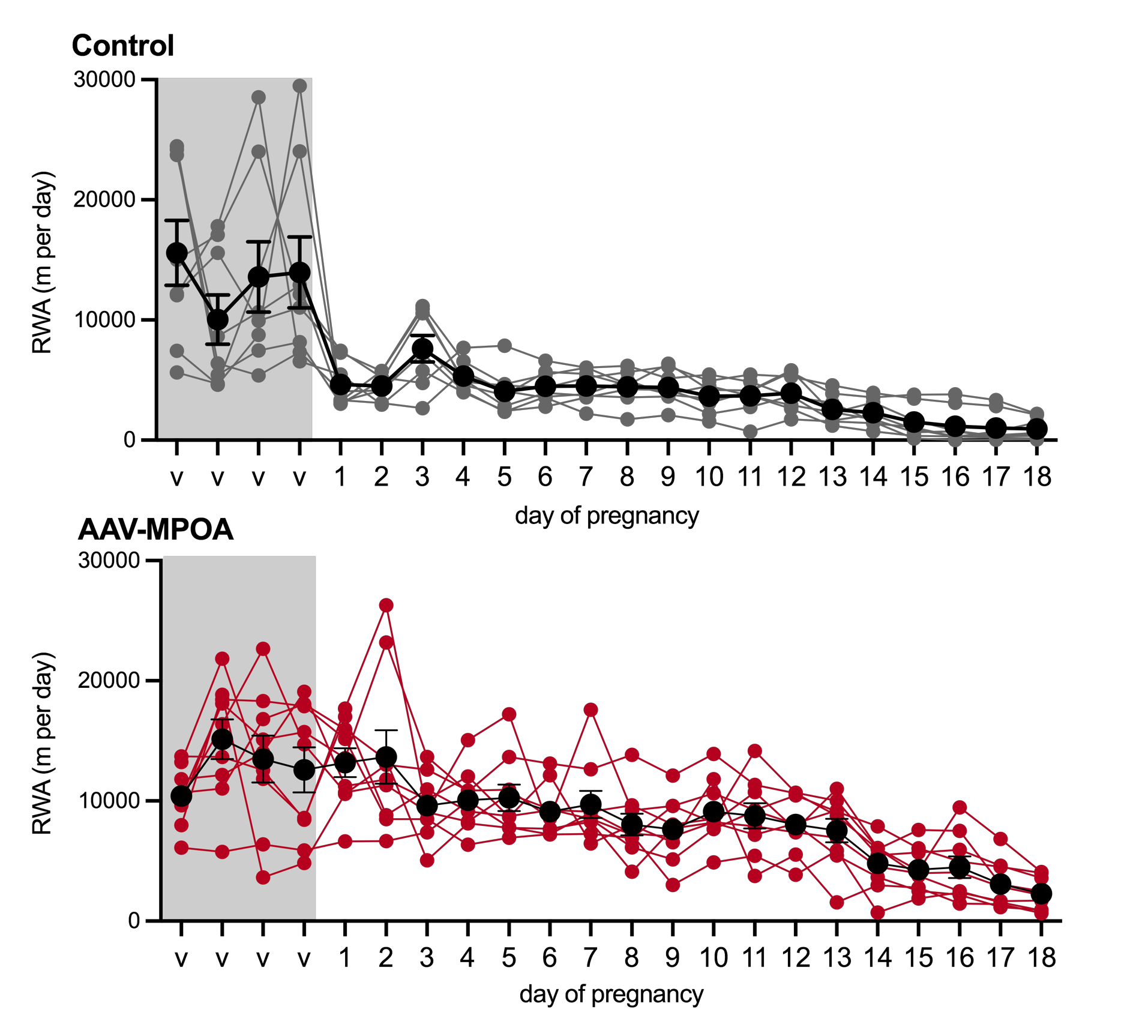

Supplement: Figure 6—source data 1. — Black lines show the mean ± SEM for the group. [file elife-62260-fig6-data1.tif]
