# Supplementary material for: A reduction in voluntary physical activity in early pregnancy in mice is mediated by prolactin
Source: eLife. 2021 Sep 16;10:e62260. doi: 10.7554/eLife.62260 (PMC8480982; doi:10.7554/eLife.62260)
Supplement: Transparent reporting form [file elife-62260-transrepform1.docx]

***eLife’s* transparent reporting form**

We encourage authors to provide detailed information *within their submission* to facilitate the interpretation and replication of experiments. Authors can upload supporting documentation to indicate the use of appropriate reporting guidelines for health-related research (see [EQUATOR Network](http://www.equator-network.org/%20)), life science research (see the [BioSharing Information Resource](https://biosharing.org/)), or the [ARRIVE guidelines](http://www.plosbiology.org/article/info:doi/10.1371/journal.pbio.1000412) for reporting work involving animal research. Where applicable, authors should refer to any relevant reporting standards documents in this form.

If you have any questions, please consult our Journal Policies and/or contact us: [editorial@elifesciences.org](mailto:editorial@elifesciences.org).

**Sample-size estimation**

- You should state whether an appropriate sample size was computed when the study was being designed
- You should state the statistical method of sample size computation and any required assumptions
- If no explicit power analysis was used, you should describe how you decided what sample (replicate) size (number) to use

Please outline where this information can be found within the submission (e.g., sections or figure legends), or explain why this information doesn’t apply to your submission:

No explicit power analysis was used to determine group size. Group sizes were based on our previous work examining energy homeostasis during pregnancy.

**Replicates**

- You should report how often each experiment was performed
- You should include a definition of biological versus technical replication
- The data obtained should be provided and sufficient information should be provided to indicate the number of independent biological and/or technical replicates
- If you encountered any outliers, you should describe how these were handled
- Criteria for exclusion/inclusion of data should be clearly stated
- High-throughput sequence data should be uploaded before submission, with a private link for reviewers provided (these are available from both GEO and ArrayExpress)

Please outline where this information can be found within the submission (e.g., sections or figure legends), or explain why this information doesn’t apply to your submission:

The number of times each experiment was performed, definition of biological replication for this study and group sizes are found in the methods, figure legends and on the figures themselves. Outliers were tested for using Rout outlier test (using Prism software) and the exclusion of an outlier in figure 3G has been indicated in the figure legend. Exclusion/inclusion information can be found in the methods section, in particular in sub-sections ‘stereotaxic injection of AAV’ and ‘Running wheel activity’.

**Statistical reporting**

- Statistical analysis methods should be described and justified
- Raw data should be presented in figures whenever informative to do so (typically when N per group is less than 10)
- For each experiment, you should identify the statistical tests used, exact values of N, definitions of center, methods of multiple test correction, and dispersion and precision measures (e.g., mean, median, SD, SEM, confidence intervals; and, for the major substantive results, a measure of effect size (e.g., Pearson's r, Cohen's d)
- Report exact p-values wherever possible alongside the summary statistics and 95% confidence intervals. These should be reported for all key questions and not only when the p-value is less than 0.05.

Please outline where this information can be found within the submission (e.g., sections or figure legends), or explain why this information doesn’t apply to your submission:

Statistical tests used can be found in the methods section under ‘Statistical analysis’ and throughout the results section and figure legends. P values are indicated in the results section alongside each result and in many of the figures.

(For large datasets, or papers with a very large number of statistical tests, you may upload a single table file with tests, Ns, etc., with reference to sections in the manuscript.)

**Group allocation**

- Indicate how samples were allocated into experimental groups (in the case of clinical studies, please specify allocation to treatment method); if randomization was used, please also state if restricted randomization was applied
- Indicate if masking was used during group allocation, data collection and/or data analysis

Please outline where this information can be found within the submission (e.g., sections or figure legends), or explain why this information doesn’t apply to your submission:

For studies using transgenic mouse lines, allocation into experimental groups was based on genotype. For studies using C57B6/J mice, mice were randomly assigned either prolactin or vehicle treatment, or to receive prolactin or vehicle first (for counter-balanced design). To the best of our ability this was done in a manner to allow both prolactin and vehicle treated mice tested on the same day, however this was not always possible due to only carrying out the experiments on one day of the estrous cycle. Masking was carried out during data collection and data analysis, but not during group allocation for the prolactin/vehicle injections due to trying to carrying out prolactin and vehicle treatments on the same day.

**Additional data files (“source data”)**

- We encourage you to upload relevant additional data files, such as numerical data that are represented as a graph in a figure, or as a summary table
- Where provided, these should be in the most useful format, and they can be uploaded as “Source data” files linked to a main figure or table
- Include model definition files including the full list of parameters used
- Include code used for data analysis (e.g., R, MatLab)
- Avoid stating that data files are “available upon request”

Please indicate the figures or tables for which source data files have been provided:

We have provided data source files for our key data, Fig 4E and J, Fig 5C and Fig 6D. These source files show the total levels of running wheel activity for each mouse and mean of the group in graphical form to complement our figures that demonstrate behaviour changes as a ratio of initial behaviour. We also have included a spreadsheet with the raw data for these key figures.
